# Facile Synthesis of PEGylated Gold Nanoparticles for Enhanced Colorimetric Detection of Histamine

**DOI:** 10.1021/acsomega.3c10050

**Published:** 2024-03-13

**Authors:** Jahor Omping, Romnick Unabia, Renzo Luis Reazo, Melbagrace Lapening, Ryan Lumod, Archie Ruda, Rolen Brian Rivera, Noel Lito Sayson, Felmer Latayada, Rey Capangpangan, Gerard Dumancas, Roberto Malaluan, Arnold Lubguban, Gaudencio Petalcorin, Arnold Alguno

**Affiliations:** † Research Center for Energy Efficient Materials (RCEEM), Premier Research Institute of Science and Mathematics (PRISM), Mindanao State University-Iligan Institute of Technology, 9200 Iligan City, Philippines; ‡ Department of Physics, Mindanao State University-Iligan Institute of Technology, 9200 Iligan City, Philippines; § Department of Chemistry, Caraga State University, Butuan City 8600, Philippines; ∥ Department of Physical Sciences and Mathematics, College of Marine and Allied Sciences, Mindanao State University at Naawan, Naawan 9023, Misamis Oriental, Philippines; ⊥ Department of Chemistry, Loyola Science Center, The University of Scranton, Scranton, Pennsylvania 18510, United States; # Center for Sustainable Polymers, MSU-Iligan Institute of Technology, Iligan City 9200, Philippines; ¶ Department of Mathematics and Statistics, Mindanao State University-Iligan Institute of Technology, 9200 Iligan City, Philippines

## Abstract

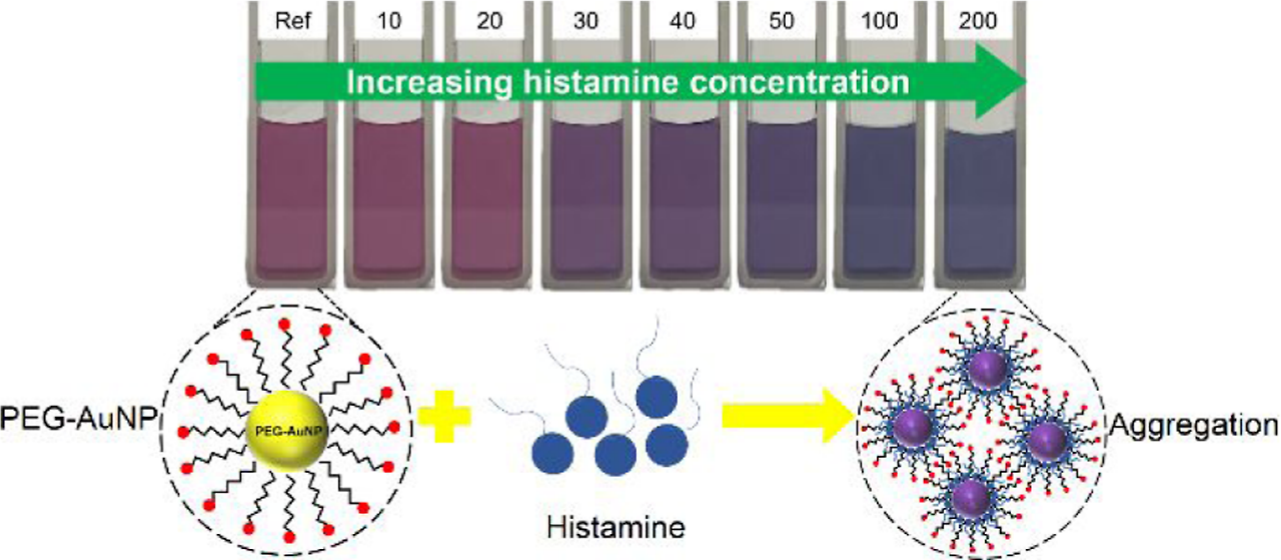

Histamine is among the biogenic amines that are formed during the microbial decarboxylation of amino acids in various food products, posing a significant threat to both food safety and human health. Herein, we present a one-step synthesis of PEGylated gold nanoparticles (PEG-AuNPs) for rapid, simple, and cost-effective colorimetric histamine detection. PEG-AuNPs’ surface plasmon resonance (SPR) range at 520–530 nm with a hydrodynamic size distribution of 20–40 nm. Fourier transform infrared (FT-IR) spectra confirmed the reduction of AuNPs at 1645 cm^–1^ along with the other observed peaks at 2870, 1350, and 1100 cm^–1^ as a strong evidence for the presence of PEG. Upon the addition of histamine to the PEG-AuNP solution, transmission electron microscopy (TEM) highlighted the aggregation of nanoparticles. In addition, red shifting and a decrease in the absorbance of the SPR peak along with the appearance of an additional peak at ∼690 nm was observed in the PEG-AuNP absorption spectra in the presence of histamine. Increasing the PEG concentration in the gold colloids leads to the formation of a protective barrier around the surface of nanoparticles, which influences the colloidal stability by impeding the aggregation of PEG-AuNPs upon histamine addition. The minimum colorimetric response of PEG-AuNPs to histamine concentration is 30 ppm, as assessed by the naked eye. The absorption ratio (*A*_690_/*A*_526_) showed a linear dynamic range from 20 to 100 ppm with a limit of detection of 9.357 μM. Additionally, the assay demonstrates a commendable selectivity toward histamine analyte.

## Introduction

Biogenic amines (BAs) are basic nitrogenous compounds that are produced during food spoilage through the decarboxylation of amino acids in high-protein foods, forming various biological amines such as histamine, tyramine, cadaverine, and putrescine.^1,2^ The excessive intake of BAs can cause toxicological reactions, such as vomiting, poisoning, headache, and breathing disorder. Among them, it was reported that high levels of histamine in food products lead to adverse effects on human health, such as food poisoning.^3−5^ Furthermore, food’s histamine levels significantly increase during improper food processing, storage time, and transportation.^6^ As a result, the detection of BAs is of utmost interest for food safety and quality control. On the other hand, low concentrations of BAs may form during storage. They occur naturally in food, particularly in fish, meat, and beverages such as wine and beer, making them a biomarker of freshness and hygiene during storage.^7^

Over the years, several techniques have been established for quantifying the amount of BAs in food, such as high-performance liquid chromatography (HPLC)^8^ and thin-layer chromatography (TLC), or gas chromatography with mass spectrometry (GC–MS).^9,10^ Although these methods are sensitive, they often require highly specialized equipment, prolonged treatment time, highly trained personnel to operate the equipment, and high-quality solvents, making them expensive and time-consuming. Hence, a new technique is needed for the swift and sensitive detection of BAs.

Recently, there has been a significant focus on the advancement of nanosensor technology because of its high sensitivity, rapid response, and ability to detect various analytes.^11,12^ Among the various metallic nanoparticles explored for sensor applications, gold nanoparticles (AuNPs) have emerged as a promising candidate for environmental and biological samples due to their selective, low-cost, and remarkable optical properties for analysis.^13−15^ By exploiting the strong surface plasmon resonance (SPR) of AuNPs, a colorimetric assay based on AuNPs has been developed since the SPR peak undergoes a shifting and broadening as a result of color change due to the aggregation and dispersion of colloidal particles;^16^ thus, a colorimetric assay based on AuNPs can be utilized as an indicator of the presence or absence of target analytes.^17,18^ Several literature studies have reported that the aggregation of AuNPs can be used to detect analytes such as histamine.^19,20^ The prominent colorimetric changes and its advantage of an easy-to-use platform pave the way for in-field analysis. However, these approaches involve sequential processes in functionalizing their sensor in response to target analytes and require a long incubation time during reaction.

To address this gap, one promising approach is the introduction of poly(ethylene glycol) (PEG) to the AuNPs. By far, the most common approach involves a place–exchange reaction, where PEG displaces the initial capping ligands of the AuNPs. Consequently, a two-step process is required to obtain PEGylated gold nanoparticles (PEG-AuNPs), such as reduction of gold precursors, followed by choice of functionalization. However, Stiufiuc et al.^21^ reported a one-step formulation of PEG-AuNPs using PEG with different molecular weights as both the reducing and stabilizing agents. Nonetheless, comprehensive investigations into the effects of varying PEG concentrations remain limited, and the existing literature lacks substantial coverage of the innovative application of PEG-AuNPs as colorimetric sensors for BA detection.

## Experimental Section

### Materials

Chloroauric acid (HAuCl_4_), PEG (Mw = 1000 g/mol), histamine, cadaverine, putrescine, and inosine were obtained from Sigma-Aldrich (Germany). Sodium hydroxide was purchased from HiMedia Laboratories (Kennett Square, USA). Ultrapure water purified with a Millipore system (18.2 MΩ) (Merck, Germany) was used as a solvent in all solutions.

### Preparation of PEGylated Gold Nanoparticles

PEG-AuNPs were synthesized following a previously reported method with slight modifications.^21^ Briefly, precise temperature control was achieved by employing a water bath. An Erlenmeyer flask containing a mixture of unmodified PEG, NaOH (1%), and ultrapure water was heated to 50 °C with constant and vigorous stirring by using a magnetic stirrer. This sample is labeled as PEG_normal_. Subsequently, an aqueous solution of HAuCl_4_ was promptly added to the PEG solution. The resulting mixture was gradually heated to 80 °C. The formation of PEG-AuNPs was confirmed by the appearance of a distinctive ruby-red color. Different volumes of PEG1000 (i.e., 170, 680, and 1020 μL labeled as PEG_half_, PEG_double_, and PEG_triple_, respectively) were added into the reaction while keeping the quantities and concentrations of HAuCl_4_ and NaOH constant. Subsequently, the resulting mixtures were cooled to room temperature and stored at 4 °C for further use.

### Colorimetric Test with Histamine Solution

The analyte standard solutions were prepared by dissolving varying amounts of histamine in ultrapure water to achieve concentrations of 10, 20, 30, 40, 50, 100, and 200 ppm. To conduct colorimetric testing, 50 μL of the analyte solution was introduced into 2 mL of the PEG-AuNP solution and was allowed to react for 30 s to 1 min. The resulting mixture was photographed to observe any changes in color with an increasing histamine concentration. Then, characterization with UV–vis spectroscopy was carried out to assess changes in the SPR of AuNPs in response to their interaction with histamine. It is important to note that all solutions were freshly prepared and all experiments were performed at room temperature.

### Measurement and Characterization

UV–visible spectra of PEG-AuNPs and the interaction with histamine analyte were acquired using a Thermo Scientific GENESYS 10S instrument (Massachusetts, USA). In each measurement, 2 mL of the PEG-AuNP solution was dispensed into a glass cuvette and scanned across a spectral range spanning from 200 to 1000 nm with a spectral resolution of 1.8 nm. The gold colloids’ hydrodynamic size and size distribution were determined using a NANOTRAC WAVE II Analyzer (Pennsylvania, USA). For this analysis, 1 mL of each sample was introduced into a fixed cell, and the system was allowed to run for 60 s to assess particle size. Morphological examinations of both PEG-AuNPs and PEG-AuNPs in the presence of histamine was conducted by high-resolution transmission electron microscopy (TEM) and TEM-EDS using a JEM 2100Plus instrument. Fourier transform infrared spectroscopy (FT-IR) was performed at room temperature using an IRTracer-100 instrument. A small volume (3 mL) of the PEGylated gold colloids was centrifuged at 16,000 rpm for 10 min to remove excess PEG from the solution. The supernatant was then decanted, and a small amount (droplet) of the precipitate was carefully deposited onto the crystal of the spectrometer, allowing it to air-dry for analysis.

## Results and Discussion

**Figure 1:**
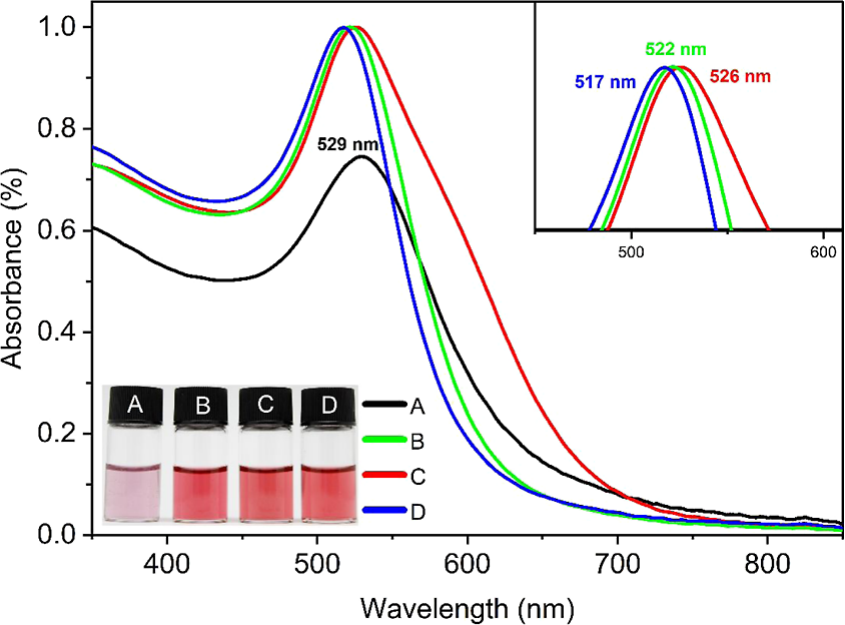
UV–vis spectra of PEG-AuNPs with varying amounts of PEG^1000^: (black dash) PEG_half_, (red dash) PEG_normal_, (green dash) PEG_double_, and (blue —dash) PEG_triple_. The inset (lower left) displays the actual photographs of PEG-AuNPs and (upper right) the zoomed-out region of the SPR peaks of PEG-AuNPs showing a blue-shift upon increasing PEG concentration.

This study prepared a AuNP colloidal solution through a one-step process using PEG as both the reducing and stabilizing agent, which exhibited a characteristic ruby-red color (see inset of Figure 1) having a SPR peak at λ_max_ = 526 nm for spherical AuNPs, as shown in Figure 1.^22^ It is noteworthy that the λ_max_ for spherical AuNPs typically falls within the range of 500–550 nm, whereas nonspherical nanoparticles exhibit an additional peak in the range of 600–700 nm.^23,24^ The SPR depends on various factors, including particle size, shape, interparticle distance, and the surrounding environment.^25,26^

During synthesis, reducing the volume of added PEG by half resulted in the broadening of the SPR peak and a red shift of λ_max_ to 529 nm, indicating the formation of larger-sized AuNPs (Figure 1, black curve). On the other hand, increasing the volume of the added PEG precursor led to a narrow SPR peak of AuNPs and blue-shifted to 517 nm λ_max_, which can be attributed to the formation of smaller PEG-AuNPs.

**Figure 2:**
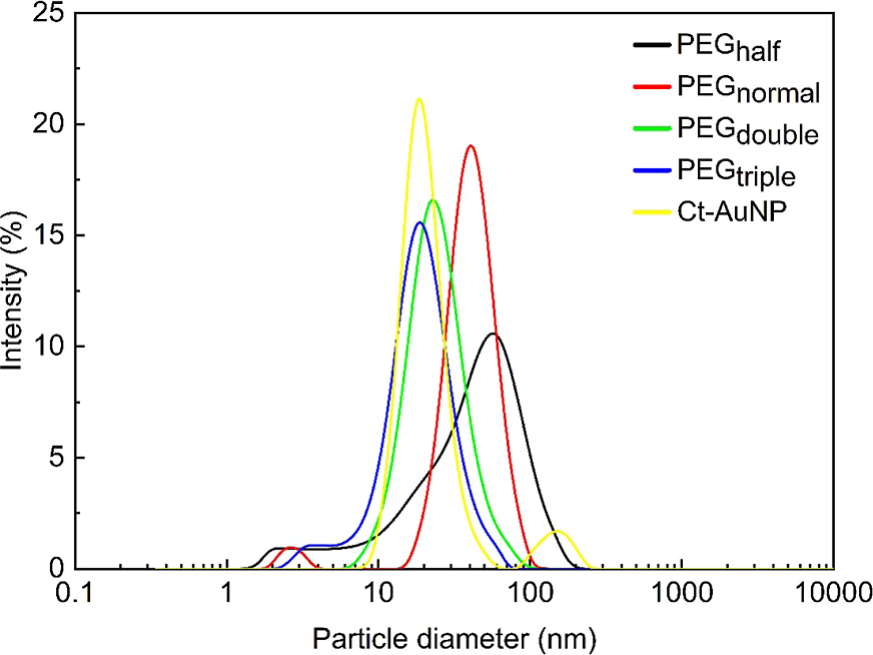
Size distribution of Ct-AuNP and PEG-AuNPs with varying amounts of PEG^1000^: (black dash) PEG_half_, (red dash) PEG_normal_, (green dash) PEG_double_, and (blue dash) PEG_triple_. The increase in the PEG concentration resulted in a decrease in hydrodynamic sizes of PEG-AuNP.

To assess the dimensions and dispersion of AuNPs in the colloidal solution, DLS measurements were employed. Figure 2 shows the size distributions of both the citrate-reduced and PEG-AuNPs, providing a basis for comparison. Both citrate-reduced AuNPs (Ct-AuNP) and PEG-AuNPs exhibit hydrodynamic sizes within the nanometer-scale range, i.e., 17 nm for bare Ct-AuNP and 37 nm for PEG-AuNP (PEG_normal_). The difference in the hydrodynamic diameter is a consequence of the PEG attachment onto the surface of nanoparticles, forming a “brush-like” layer,^27^ which augments the apparent hydrodynamic diameter measured. Consequently, further variation of the PEG volume in the colloidal solution led to a reduction in the hydrodynamic diameter, from 37 nm for PEG_normal_ to 17 nm for PEG_triple_ (see Table 1). This phenomenon can be attributed to the fact that smaller particles offer a higher surface curvature. By increasing the volume of PEG, more polymers are loaded onto the nanoparticle, thereby inhibiting nanoparticle coagulation and subsequently decreasing the hydrodynamic diameter of the AuNPs.^28^ Meanwhile, decreasing the PEG concentration by half leads to a broad size distribution with an average hydrodynamic diameter of 40 nm, corroborating the findings obtained from the UV–vis spectra.

**Table 1:** Size and Absorption Maximum Values of Colloidal AuNPs Reduced with Different Concentrations of PEG

PEGylated nanoparticles	PEG concentration (μmol)	size	absorption maximum (nm)
PEG_half_	204	40	529
PEG_normal_	408	37	526
PEG_double_	816	21	522
PEG_triple_	1224	19	517
Ct-AuNP	0	17	518

Conversely, PEG plays a dual role during the synthesis process. First, it acts as a reducing agent for gold precursor ions, facilitating the nucleation and growth of AuNPs. Simultaneously, PEG attaches to the surface of AuNP, creating a steric barrier that ensures colloidal stability and prevents particle aggregation.^29^ However, the amount of PEG in the colloidal solution can significantly influence the formation of AuNPs, i.e., a minimal amount of PEG employed in the synthesis process leads to the formation of larger and polydisperse particles due to limited availability of PEG molecules for the reducing and stabilizing process. Additionally, the presence of NaOH in the preparation method provides an alkaline environment within the solution, which promotes the rapid formation of AuNPs.^30^

**Figure 3:**
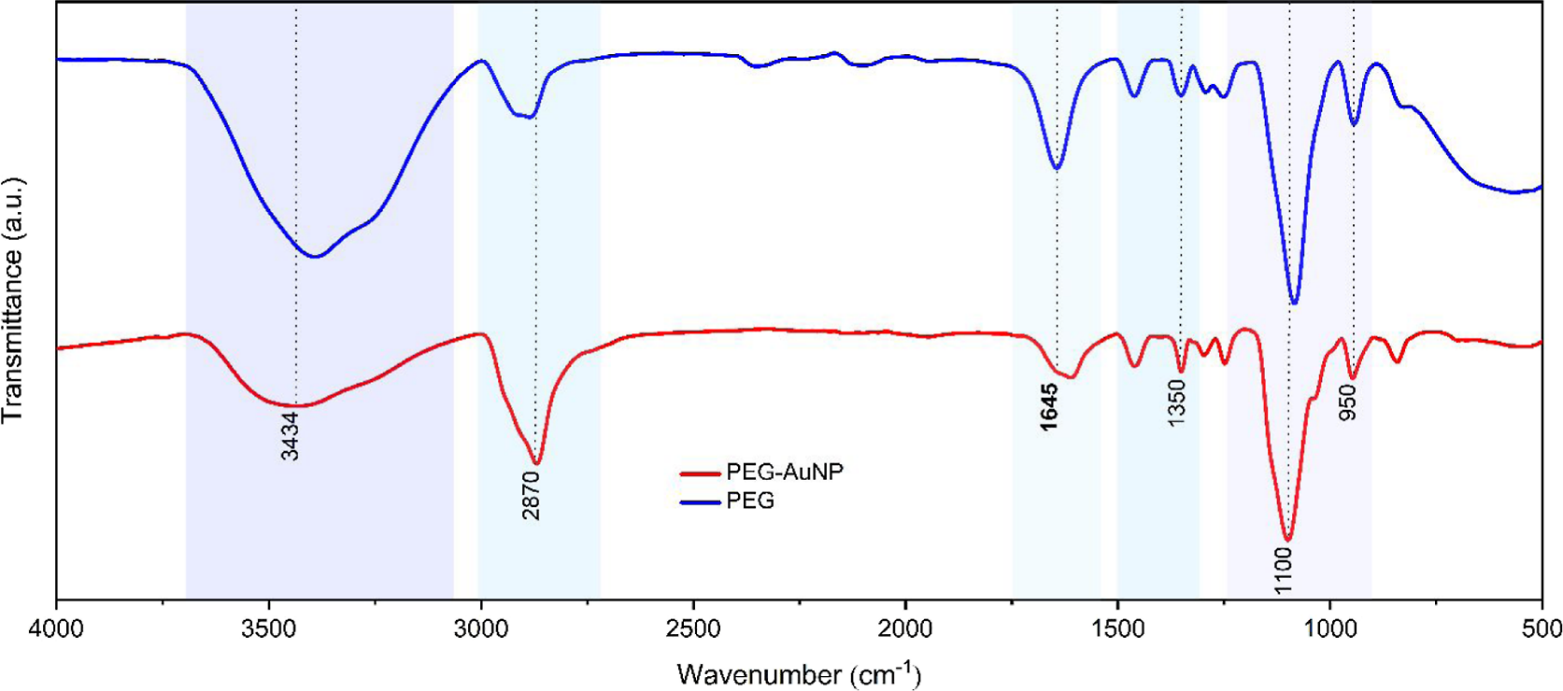
FTIR spectra of PEG1000 (blue line) and PEG-AuNPs (red line). All of the distinctive functional groups of PEG molecules attached to the gold nanoparticles are clearly visible, implying successful reduction.

To verify the attachment of PEG molecules to AuNPs, FTIR spectroscopy was used. Figure 3 displays the FTIR spectra of PEG-AuNP and pure polymer PEG. Since PEG is a derivative of ethylene glycol, a broad and prominent peak is observed located at 3434 cm^–1^, corresponding to the O–H stretching of the hydroxyl group within the polymer. Additionally, the presence of methylene C–H stretching is evident at 2870 cm^–1^, along with observable bending modes such as O–C–H, C–C–H, and C–O–H angles at approximately 1350 cm^–1^. The C–O–C stretch band characteristics of ether appear at 1100 cm^–1^, complemented by the −CH– out-of-plane bending vibrations at around 946 cm^–1^. These observed peaks serve as strong evidence for the presence of PEG in the spectra. Meanwhile, the presence of a vibrational peak at 1645 cm^–1^ was attributed to the asymmetric stretching vibrational mode of the gold carboxylate unidentate bond observed in the spectra of PEG-AuNPs. This observation suggests that PEG undergoes an oxidative transformation during the formation process, converting terminal alcohol groups into carboxylate entities. This transformation accounts for the negative charge on the surface of gold colloids and their interaction with PEG, further corroborating that PEG functions as both the reducing and stabilizing agent.^31^

**Figure 4:**
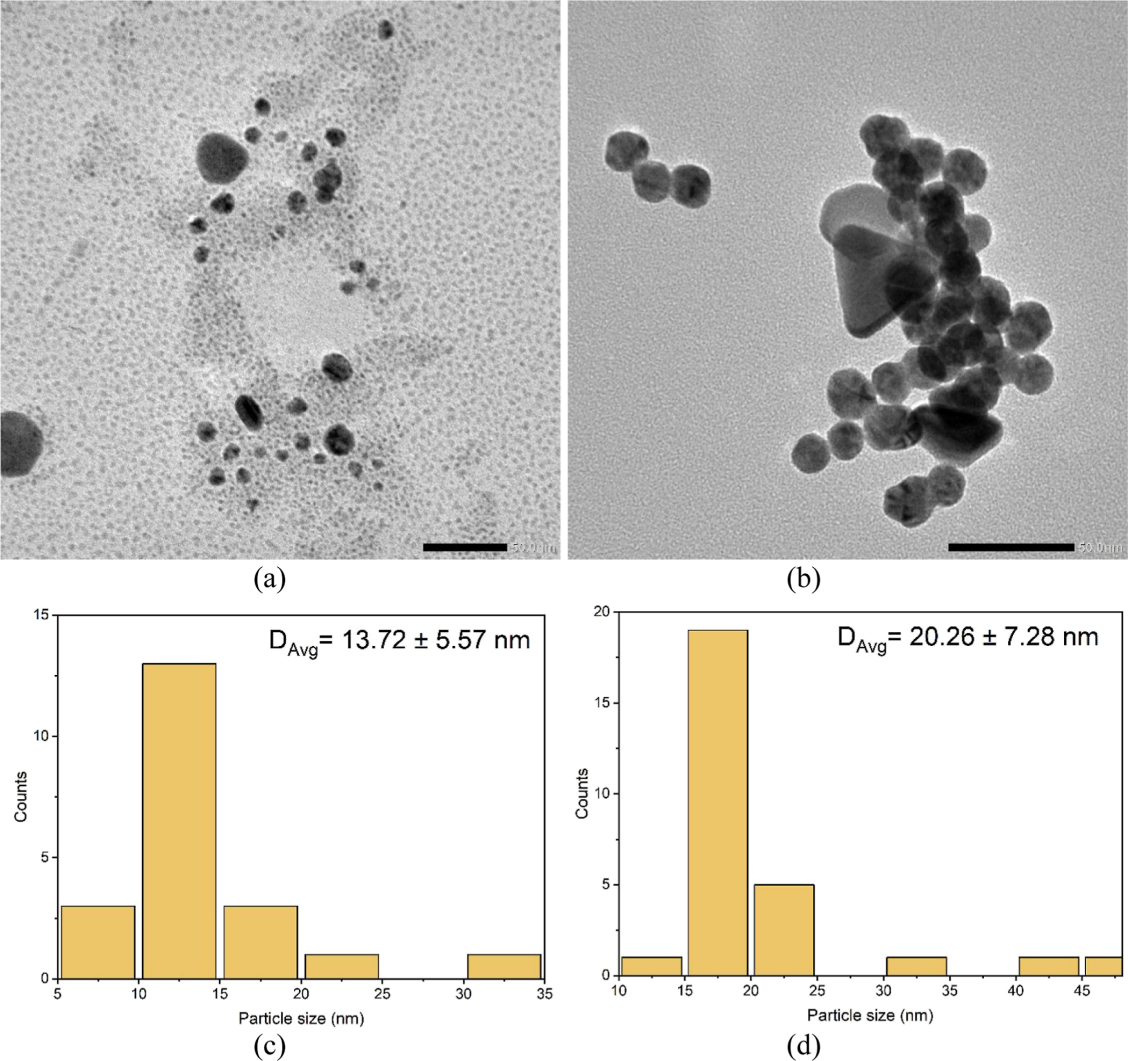
TEM micrographs of (a) PEG-capped AuNP and (b) PEG-AuNPs in the presence of histamine (100 ppm). The scale bars are 50 nm. Particle size statistics of the (c) PEG-capped AuNP and (d) PEG-AuNPs with histamine (100 ppm).

Now, standard histamine testing was conducted to assess the performance of the PEG-AuNPs for colorimetric detection. The TEM images of gold colloids shown in Figure 4a confirm the predominance of spherical AuNPs with an average particle size of 13.72 ± 5.57 nm. While spherical shapes are prevalent, certain particles exhibit facets, a characteristic arising from their nanocrystalline nature.^32^ Addition of histamine to the PEG-AuNP solution leads to the aggregation of nanoparticles, as shown in Figure 4b, indicated by the structural and colloidal properties of AuNPs. The particle size distribution of PEG-AuNPs reveals significant increase in particle size statistics with the presence of histamine analyte.

**Scheme 1:**
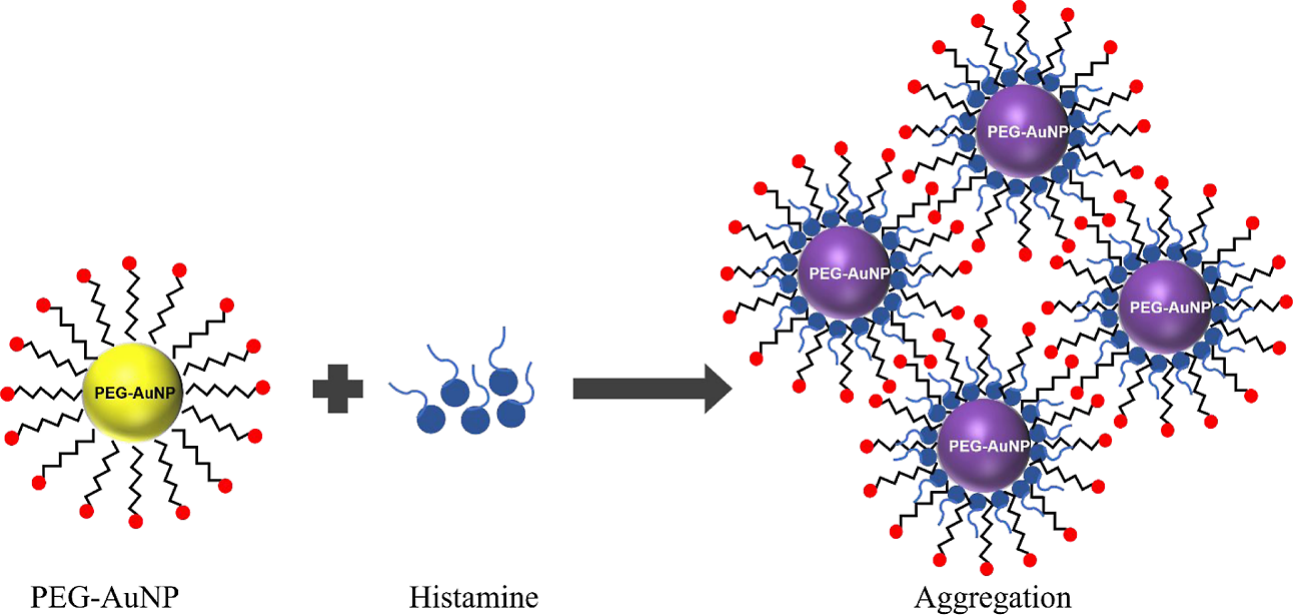
Proposed Mechanism for Histamine Detection via PEG-AuNPs The presence of histamine analyte induces the aggregation of PEG-AuNPs.

To understand the aggregation mechanism of PEG-AuNP upon interaction with histamine, this study proposed a mechanism, as illustrated in Scheme 1. The histamine molecule comprises an imidazole ring and an aliphatic amino group, which present as reaction sites. It is worth noting that imidazole has a well-documented strong affinity to AuNPs.^6,33^ Here, imidazole’s nucleophilic nature, which attracts positively charged atoms, facilitates its penetration to the surface of AuNPs, thereby inducing aggregation. On the other hand, although the aliphatic amino group of histamine does not exhibit an inherent affinity for AuNPs,^34^ the interaction between the oxygen atoms on the surface of AuNP and the protonated aliphatic amino group of histamine promotes aggregation via hydrogen bonding. This interaction encourages the proximity of the imidazole ring to the AuNP.

**Figure 5:**
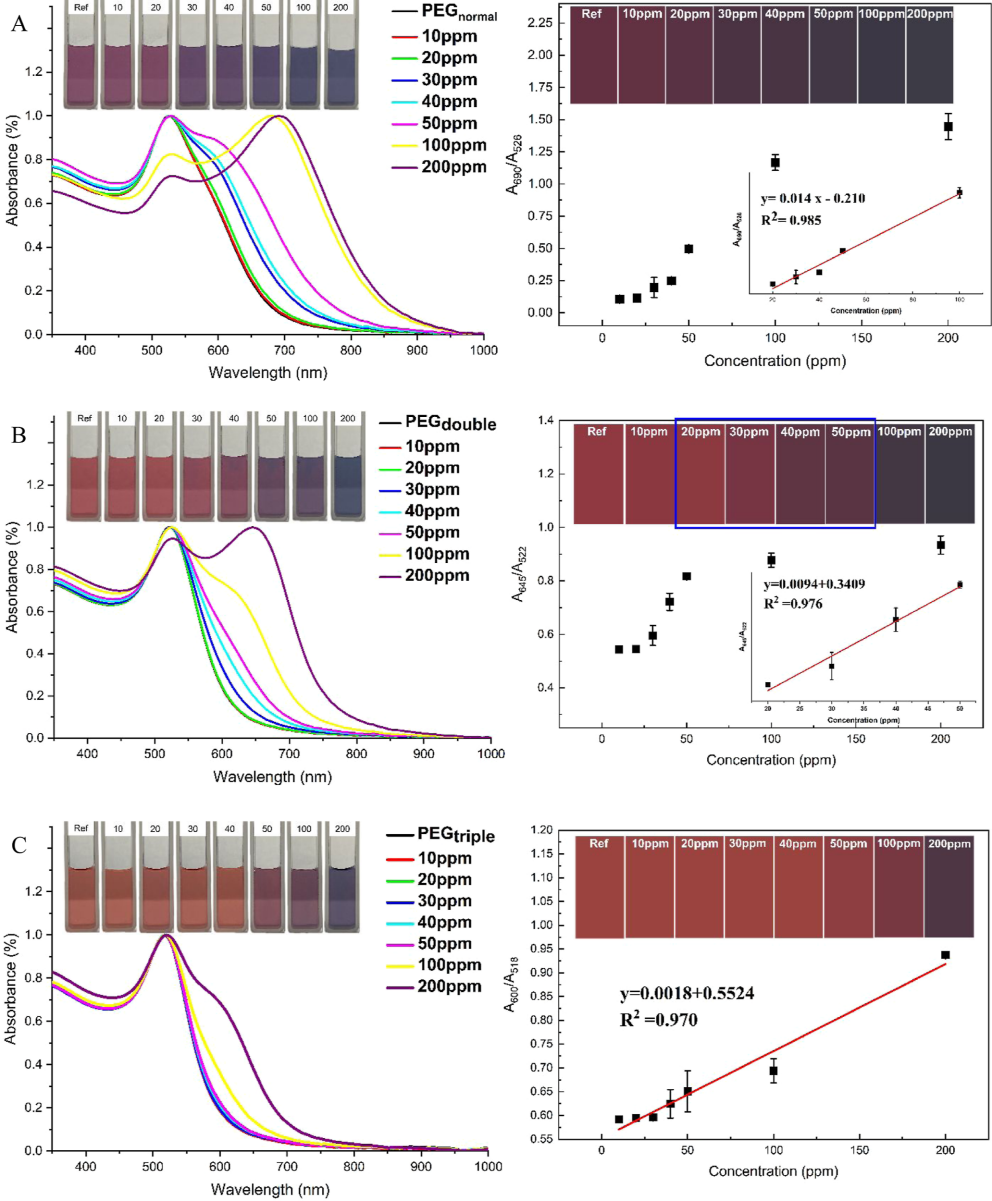
(Left) Absorption spectra of PEG-AuNPs solutions after the addition of different concentrations of histamine: (A) PEG_normal_, (B) PEG_double_, and (C) PEG_triple_. (Right) Corresponding absorption ratios of PEG-AuNPs with increasing histamine concentration: reference, 10, 20, 30, 40, 50, 100, and 200 ppm. The sensitivity of PEG-AuNPs toward histamine decreases with an increase in the PEG concentration. The inset displays the actual solution and actual chroma images.

Furthermore, the localized SPR around 520 nm in the absorption spectra, a characteristic peak of AuNPs, is highly sensitive to surface modifications resulting from interactions with analytes or contaminants.^35^ It should be noted that we opt not to include the PEG_half_ in the analyte testing due to its polydisperse nature and relatively large hydrodynamic size (as summarized in Table 1) to avoid complication in the analysis upon histamine addition. Figure 5A–C illustrates the absorption spectra of PEG-AuNPs colloids added with different histamine concentrations ranging from 10 to 200 ppm, in accordance with the guidelines set by the US FDA and FAO/WHO.^36^ In their dispersed state in a colloidal solution, AuNPs typically exhibit a wine-red color visible to the naked eye. However, when AuNPs aggregate, they undergo a noticeable color shift toward a purplish-blue hue.^7,17,37^ This observation was depicted in the PEG-AuNP solution upon interaction with the histamine analyte, as shown in Figure 5A (see inset). Within a time frame of 30 s to 1 min following interaction with the target analyte, the solution completely transformed into a purplish-blue color. This naked-eye observation of color transition was validated by attenuation in the absorption spectra of the PEG-AuNP solution showing the gradual decrease in the SPR peak intensity of PEG-AuNPs at ∼526 nm coupled with the emergence of a shoulder peak around 600 nm for PEG_normal_ solution containing 30 ppm of histamine. As histamine concentration reaches 200 ppm, PEG-AuNP solutions displayed a distinctive color change from purplish-blue to dark blueish hue along with shifting of the SPR peak to a longer wavelength and the appearance of a 690 nm peak, signifying the aggregation of AuNPs in the presence of histamine, which is in agreement with the TEM micrograph (see Figure 4b). The histamine concentration that is visually detectable with PEG-AuNP as the colorimetric sensor was 30 ppm. Furthermore, a strong linear relationship between absorption ratio (*A*_690_/*A*_526_) and histamine concentrations within the range of 20–100 ppm is observed (Figure 5A, corresponding calibration curve). The calibration curve exhibits a good correlation coefficient (*R*^2^) of 0.985 with a limit of detection (LOD) of 9.357 μM.

Numerous studies have highlighted the critical role of AuNP size in their colorimetric response, particularly their sensitivity to aggregation. This sensitivity allows for controlled nanoparticle assembly and tunable sensor responses.^38,39^ Conversely, we conducted a comparative analysis of histamine detection using PEG_double_ and PEG_triple_ colloids (see Figure 5B,C) to assess their respective sensitivity levels. Upon the addition of histamine (>20 ppm) into the PEG-AuNP, noticeable color changes from wine-red to darker hue remained observable to the naked eye. In addition to this visual change, we observed red-shifting in the SPR around 520 nm, accompanied by the emergence of a peak at ∼630 nm. Meanwhile, as the amount of PEG in the gold colloids increased, the sensitivity of histamine detection was decreased; i.e., the detection threshold shifted from 30 ppm for PEG_normal_ to 100 ppm for PEG_triple_ (see Figure 5C). This observations may be attributed to the protective layer of PEG on the surface of AuNPs since increasing amounts of PEG introduced to the gold colloids led to higher capping density of PEG on the AuNPs, affecting the stability and sensitivity of AuNPs^40,41^ The increase of the PEG layer on the surface of AuNPs simultaneously reduced the accessibility of the imidazole ring to penetrate the surface of AuNPs, thus limiting the potential for aggregation.

**Figure 6:**
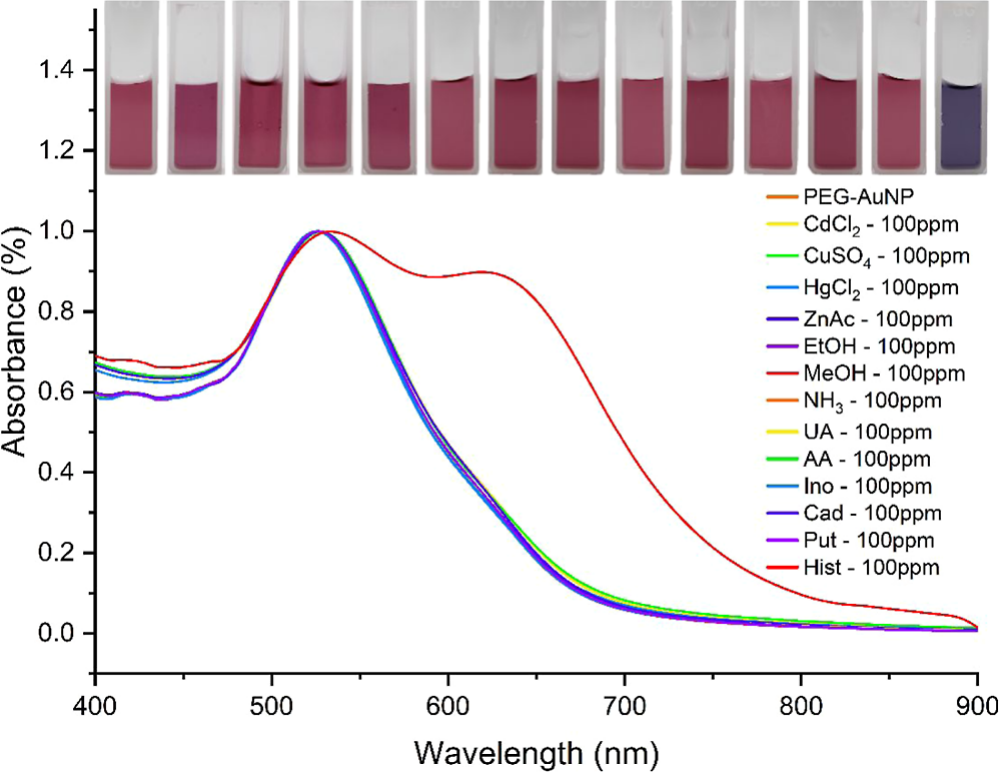
UV–vis absorption spectra of PEG-AuNPs tested with different analytes such as cadmium chloride, copper sulfate, mercury chloride, zinc acetate, ethanol, methanol, ammonia, uric acid, acetic acid, inosine, cadaverine, putrescine, and histamine. The inset displays the actual image of the solution and demonstrates that only the solution with the presence of histamine analyte changed from wine-red to bluish hue, while others remained unchanged.

The feasibility of the PEG-AuNPs sensor for histamine detection was investigated through colorimetric response with various BAs [histamine (His), cadaverine (Cad), putrescine (Put), inosine (Ino)], and other potential interfering analytes at 100 ppm. As manifested in Figure 6, none of these analytes could cause attenuation of the SPR peak of the PEG-AuNPs as histamine did. Upon the addition of histamine, the wine-red color of PEG-AuNPs gradually changed to a purplish-blue, and a notable red-shift around 520 to 600 nm was observed from the UV–vis absorption spectra, attributed to the possible aggregation of PEG-AuNPs. In addition, other organic [ethanol (EtOH), methanol (MeOH), ammonia (NH_3_), uric acid (UA), acetic acid (AA)] and inorganic compounds [cadmium chloride (CdCl_2_), copper sulfate (CuSO_4_), mercury chloride (HgCl_2_), zinc acetate (ZnAc)] were also investigated, showing negligible color change and aggregation, implying excellent selectivity of the sensor toward histamine. The inset visually confirms the distinct color transition upon histamine exposure as compared with other analytes. Hence, PEG-AuNPs can distinctively detect histamine analytes via a colorimetric approach.

Lastly, the performance of the PEG-AuNPs colorimetric sensor presented in this study was systematically compared against existing methods reported in the literature (Table 2). A study of Li et al.^19^ reported a thiolated polyethylene glycol (PEG-SH) and dopamine-functionalized sensor probe for sensitive polymerization under the presence of BAs. The sensor demonstrated a linear response range of 1–100 μg/mL with a LOD of 2.8 μg/mL for histamine, achieved within a 4 h incubation time. Choi et al.^42^ developed a carbon disulfide (CS_2_) added colloidal gold nanoparticle-based sensor for rapid and on-site detection of BAs. A linear response range of 1–1000 μM and a LOD value of 50 μM were obtained after 10 min of reaction time. Additionally, Orouji et al.^43^ designed a multicolor sensor array based on the metallization of silver ions on the surface of gold nanorods (AuNRs) and gold nanospheres (AuNSs) in the presence of BAs. The sensor exhibited a LOD of 4.79, 8.85, 10.03, 27.29, 2.46, and 14.26 μM respectively for spermine, tryptamine, ethylenediamine, tyramine, spermidine, and histamine under 10 min of incubation.

**Table 2:** Comparison of the AuNP-Based Probe for the Detection of Various Biogenic Amines

probe	analyte	linear range	response time (min)	LOD (μM)	ref
citrate-capped gold nanoparticle, functionalized with thiolated polyethylene glycol (PEG-SH) and addition of dopamine	histamine, putrescine, cadaverine, spermine, spermidine, tyramine, and tryptamine	1–100 μg/mL	4 h	25.2	(19)
citrate-reduced AuNP, functionalized with carbon disulfide (CS_2_)	cadaverine, putrescine, histamine, and tyramine	1.0 to 1000.0 μM	10	50.0	(42)
silver deposition on gold nanorods (AuNRs) and gold nanospheres (AuNSs)	spermine	20–800 μM	10	4.79	(43)
	tryptamine	40–800 μM		8.58	
	ethylenediamine	60–800 μM		10.03	
	tyramine	80–800 μM		27.29	
	spermidine	10–800 μM		2.46	
	histamine	40–800 μM		14.26	
citrate-reduced AuNP, stabilized with thiol-PEG-acid	melamine	1 nmol to 1 mmol	35	1.05 × 10^–3^	(38)
one-step PEG-AuNPs	histamine	20 to 100 ppm	<1	9.357	this work

Accordingly, our PEG-AuNP sensor exhibited a linear response range to histamine concentrations ranging from 20 to 100 ppm, with a good correlation coefficient of 0.985 (Figure 5A, corresponding calibration curve). In addition, the performance of our probe demonstrated a simple, rapid response and exceptional selectivity for histamine detection, with a detection limit of 9.357 μM within a short incubation time of less than 1 min, exceeding the majority of the previously reported methods described in Table 2.

**Figure 7:**
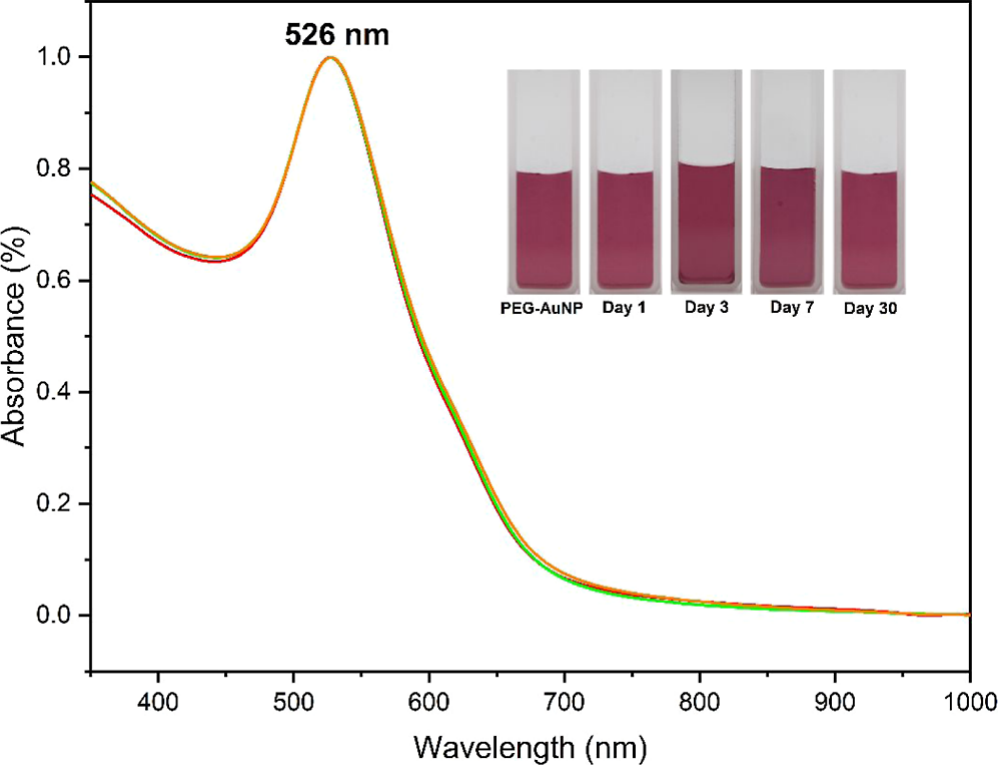
Time-dependence stability study displaying the absorbance of the SPR peak of the PEG-AuNP solution stored at 4 °C throughout various durations: (black dash) as synthesized, (red dash) 1 day, (green dash) 3 days, (blue dash) 7 days, and (yellow dash) 30 days. Inset: actual solution of the PEG-AuNP solution.

To attest to the colloidal stability of the PEG-AuNP solution for practical application, we further conducted a time-dependent stability study, monitoring the attenuation of the SPR peak and the change in color of the solution throughout extended storage durations. The absorption spectra of the PEG-AuNP stored at 4 °C for different time intervals are shown in Figure 7. It can be inferred from the graph that both the SPR peak and the color of the solution remain largely unchanged, even after increasing days of storage, implying the exceptional stability of the colloidal solution (Figure 7, inset). This remarkable stability of PEG-AuNPs can be attributed to the steric repulsion between nanoparticles. The PEG molecules are attached to AuNPs usually form hydrogen bonds with the surrounding solvent, effectively preventing aggregation over time. Hence, the PEG-AuNP sensor demonstrated sufficient stability under extended storage durations.

## Conclusions

This study successfully synthesized PEGylated-AuNPs using low concentrations of HAuCl_4_, with varying amounts of added PEG to serve as both the reducing and stabilizing agent of AuNPs. The amount of PEG added into the solution dramatically influences the production of AuNPs with ∼20 to ∼30 nm hydrodynamic diameter. By varying the amount of added PEG in the solution, PEG-AuNPs colloids display AuNPs’ characteristic SPR at ∼521 nm. Except for PEG_half_, nanoparticles displayed a high level of homogeneity in size distribution, implying that the amount of PEG plays a vital role in the formation and stabilization of AuNPs.

We also developed a rapid and cost-effective colorimetric assay designed for the detection of BAs. The strong affinity of the imidazole ring toward AuNP and the hydrogen bonding between the oxygen atoms and the aliphatic amino group of histamine (synergistically) induces aggregation. Moreover, an increase in the number of PEG molecules in the solution leads to reduced sensitivity or detection capabilities for histamine. Our assay is capable of detecting the target analyte with a remarkable LOD of 9.357 μM. Comparative analysis was also conducted for PEG_double_ and PEG_triple_, revealing that the amount of PEG molecules in the solution significantly influences the sensitivity of PEG-AuNPs in their interaction with histamine. We determined that an optimal volume of 340 μL (PEG) in the method is the optimum amount for enhanced sensitivity and selectivity for histamine detection.
